# Supplementary material for: The human insula processes both modality-independent and pain-selective learning signals
Source: PLoS Biol. 2022 May 6;20(5):e3001540. doi: 10.1371/journal.pbio.3001540 (PMC9116652; doi:10.1371/journal.pbio.3001540)
Supplement: S1 Table — Parameters obtained from linear mixed models with random subject intercept. Differences between the conditions are largest in trials with no PE and smallest in trials with modality prediction error (cf. S1 Fig). Data used to produce the table can be found at https://www.doi.org/10.17605/OSF.IO/7JBV3. PE, prediction error; SCR, skin conductance response. (DOCX) [file pbio.3001540.s001.docx]

| **Subanalysis** | **Term** | **Estimate** | **SE** | **CILower** | **CIUpper** | **p** |
| --- | --- | --- | --- | --- | --- | --- |
|  |  |  |  |  |  |  |
| **No prediction error** | **Modality** | -0.0442 | 0.0307 | -0.1044 | 0.0161 | 0.1506 |
|  | **Intensity** | 0.2444 | 0.0305 | 0.1845 | 0.3042 | 2 x 10^-15* |
|  | **Modality*Intensity** | -0.2023 | 0.0433 | -0.2873 | -0.1173 | 3 x 10^-6* |
|  |  |  |  |  |  |  |
| **Intensity prediction error** | **Modality** | -0.0506 | 0.0603 | -0.1689 | 0.0677 | 0.4014 |
|  | **Intensity** | 0.2479 | 0.0598 | 0.1305 | 0.3653 | 4 x 10^-5* |
|  | **Modality*Intensity** | -0.026 | 0.0838 | -0.1904 | 0.1385 | 0.7566 |
|  |  |  |  |  |  |  |
| **Modality prediction error** | **Modality** | -0.056 | 0.0698 | -0.1929 | 0.0809 | 0.4227 |
|  | **Intensity** | 0.1138 | 0.071 | -0.0257 | 0.2532 | 0.1096 |
|  | **Modality*Intensity** | 0.0973 | 0.0994 | -0.0977 | 0.2924 | 0.3277 |

*p < 0.001.
