# Supplementary material for: The human insula processes both modality-independent and pain-selective learning signals
Source: PLoS Biol. 2022 May 6;20(5):e3001540. doi: 10.1371/journal.pbio.3001540 (PMC9116652; doi:10.1371/journal.pbio.3001540)
Supplement: S2 Table — Data used to produce the table can be found at https://www.doi.org/10.17605/OSF.IO/7JBV3. BDI-II, Beck Depression Inventory II; exp., experiment; FPQ, Fear of Pain Questionnaire; MDMQ, Multidimensional Mood Questionnaire; PHQ15, Patient Health Questionnaire-15; PRSS, Pain-Related Self-Statements; PSQ, Pain Sensitivity Questionnaire; PVAQ, Pain Vigilance and Awareness Questionnaire; STAI, State-Trait Anxiety Inventory. (DOCX) [file pbio.3001540.s002.docx]

| **Questionnaire** | **Construct** | **Mean±SD** | **Sample range** | **Possible range** |
| --- | --- | --- | --- | --- |
| **BDI-II** [SR 1,2] | Depression | 4.0±3.7 | 0-13 | 0-63 |
| **PHQ15** [SR 3] | Somatization | 3.4±2.7 | 0-10 | 0-30 |
| **FPQ** [SR 4] |  |  |  |  |
| severe | Fear of pain | 29.9±9.6 | 10-50 | 10-50 |
| minor | Fear of pain | 16.3±5.2 | 10-34 | 10-50 |
| **PVAQ** [SR 5] | Pain vigilance and awareness | 36.2±9.9 | 9-63 | 0-80 |
| **PSQ** [SR 6] | Pain sensitivity | 43.3±16.0 | 9-80 | 0-140 |
| **PRSS** [SR 7] |  |  |  |  |
| Catastrophizing | Pain catastrophizing | 8.4±6.1 | 1-27 | 0-45, higher more catastrophizing |
| Coping | Pain coping | 31.3±6.0 | 19-43 | 0-45, higher more active coping |
| **STAI** [SR 8,9] |  |  |  |  |
| Trait | Trait anxiety | 33.1±5.9 | 23-48 | 20-80 |
| State | State anxiety (pre experiment) | 33.4±6.2 | 23-49 | 20-80 |
| **MDMQ** [SR 10] |  |  |  |  |
| GoodBad A | Mood: Good vs bad (pre exp.) | 17.3±2.0 | 12-20 | 4-24, the higher the better mood |
| AwakeTired A | Mood: Awake vs tired (pre exp.) | 14.5±2.9 | 8-20 | 4-24, the higher the more awake |
| CalmNervous A | Mood: Calm vs nervous (pre exp.) | 16.1±2.2 | 10-20 | 4-24, the higher the calmer |
| GoodBad B | Mood: Good vs bad (post exp.) | 17.2±2.0 | 11-20 | 4-24, the higher the better mood |
| AwakeTired B | Mood: Awake vs tired (post exp.) | 11.7±3.1 | 7-18 | 4-24, the higher the more awake |
| CalmNervous B | Mood: Calm vs nervous (post exp.) | 17.2±2.4 | 11-20 | 4-24, the higher the calmer |

**References**

SR 1. Beck AT, Steer RA, Brown GK. Manual for the Beck Depression Inventory-II. San Antonio, TX: 810 Psychological Corporation Press; 1996. 811

SR 2. Hautzinger M, Keller F, Kühner C. BDI-II. Beck-Depressions-Inventar. Revision. 2nd ed. 812 Frankfurt: Pearson Assessment; 2009. 813

SR 3. Kroenke K, Spitzer RL, Williams JBW. The PHQ-15: validity of a new measure for evaluating the 814 severity of somatic symptoms. Psychosom Med. 2002;64(2):258–66. 815

SR 4. McNeil DW, Rainwater AJ. Development of the Fear of Pain Questionnaire-III. J Behav Med. 816 1998;21(4):389–410. 817

SR 5. McCracken LM. "Attention" to pain in persons with chronic pain: A behavioral approach. 818 Behav Ther. 1997;28(2):271–84. doi: 10.1016/S0005-7894(97)80047-0 819

SR 6. Ruscheweyh R, Marziniak M, Stumpenhorst F, Reinholz J, Knecht S. Pain sensitivity can be 820 assessed by self-rating: Development and validation of the Pain Sensitivity Questionnaire. 821 Pain. 2009;146(1):65–74. doi: 10.1016/j.pain.2009.06.020 822

SR 7. Flor H, Behle DJ, Birbaumer N. Assessment of pain-related cognitions in chronic pain patients. 823 Behav Res Ther. 1993;31(1):63–73. 824

SR 8. Spielberger CD, Gorsuch RL, Lushene RE. Manual for the State-Trait Anxiety Inventory. Palo 825 Alto, CA: Consulting Psychologists Press; 1970. 826

SR 9. Laux L, Glanzmann P, Schaffner P, Spielberger CD. Das State-Trait-Angstinventar. Weinheim: 827 Beltz; 1981. 828

SR 10. Steyer R, Schwenkmezger P, Notz P, Eid M. Der Mehrdimensionale Befindlichkeitsfragebogen 829 (MDBF). Handanweisung. Göttingen: Hogrefe; 1997.
