# Supplementary figures and images for: The human insula processes both modality-independent and pain-selective learning signals
Source: PLoS Biol. 2022 May 6;20(5):e3001540. doi: 10.1371/journal.pbio.3001540 (PMC9116652; doi:10.1371/journal.pbio.3001540)

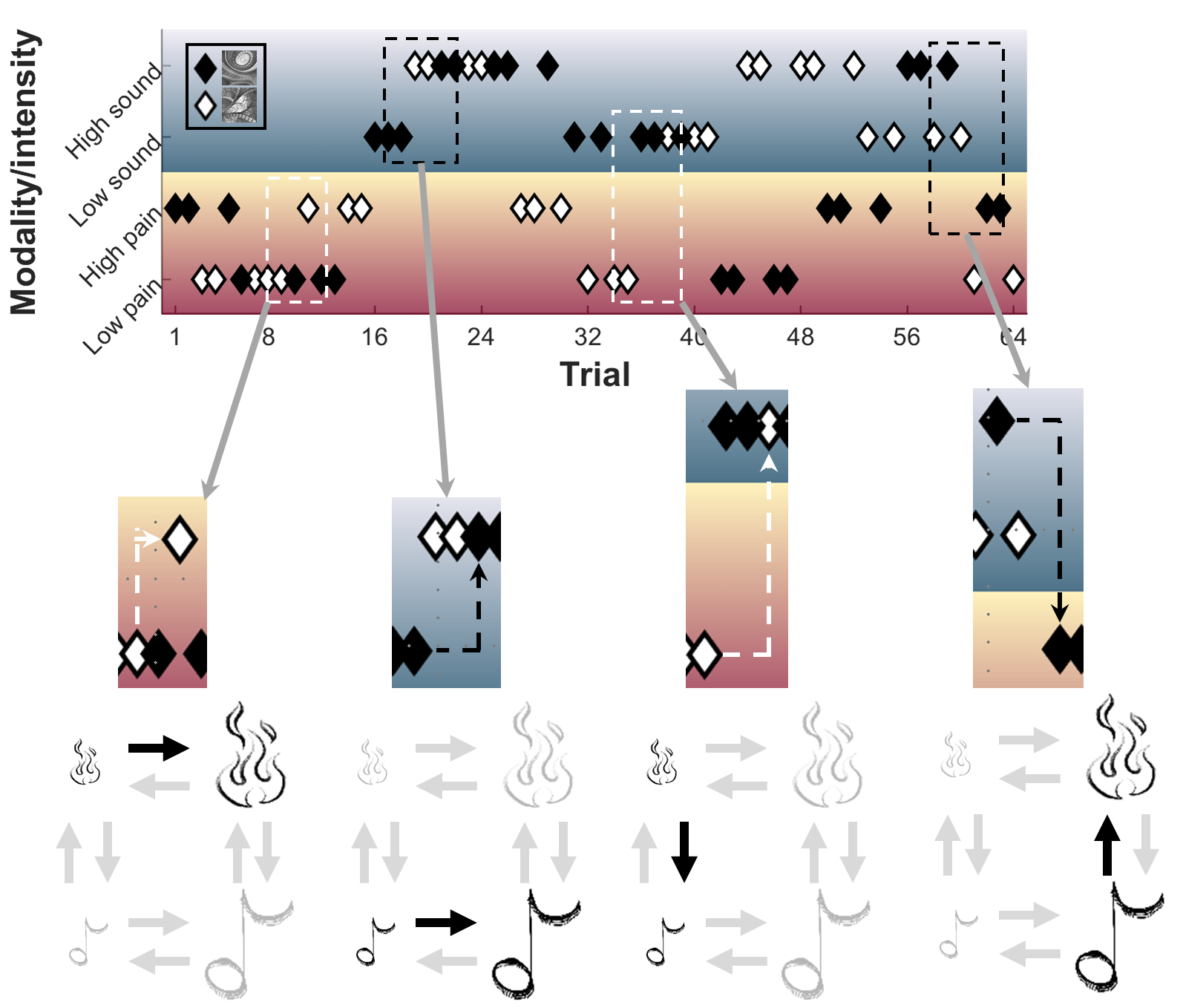

Supplement: S1 Fig — Both CS have an independent sequence of deterministic associations with one of the 4 US (also see Fig 2). The dashed lines illustrate reversals for CS1 (black) or CS2 (white). First column, CS2 intensity reversal from low to high heat; second column, CS1 intensity reversal from low to high sound; third column, CS2 modality reversal from low heat to low sound; fourth column, modality reversal from high sound to high heat. Data used to produce the figure can be found at https://www.doi.org/10.17605/OSF.IO/7JBV3. CS, conditioned stimuli; US, unconditioned stimuli. (TIF) [file pbio.3001540.s003.tif]

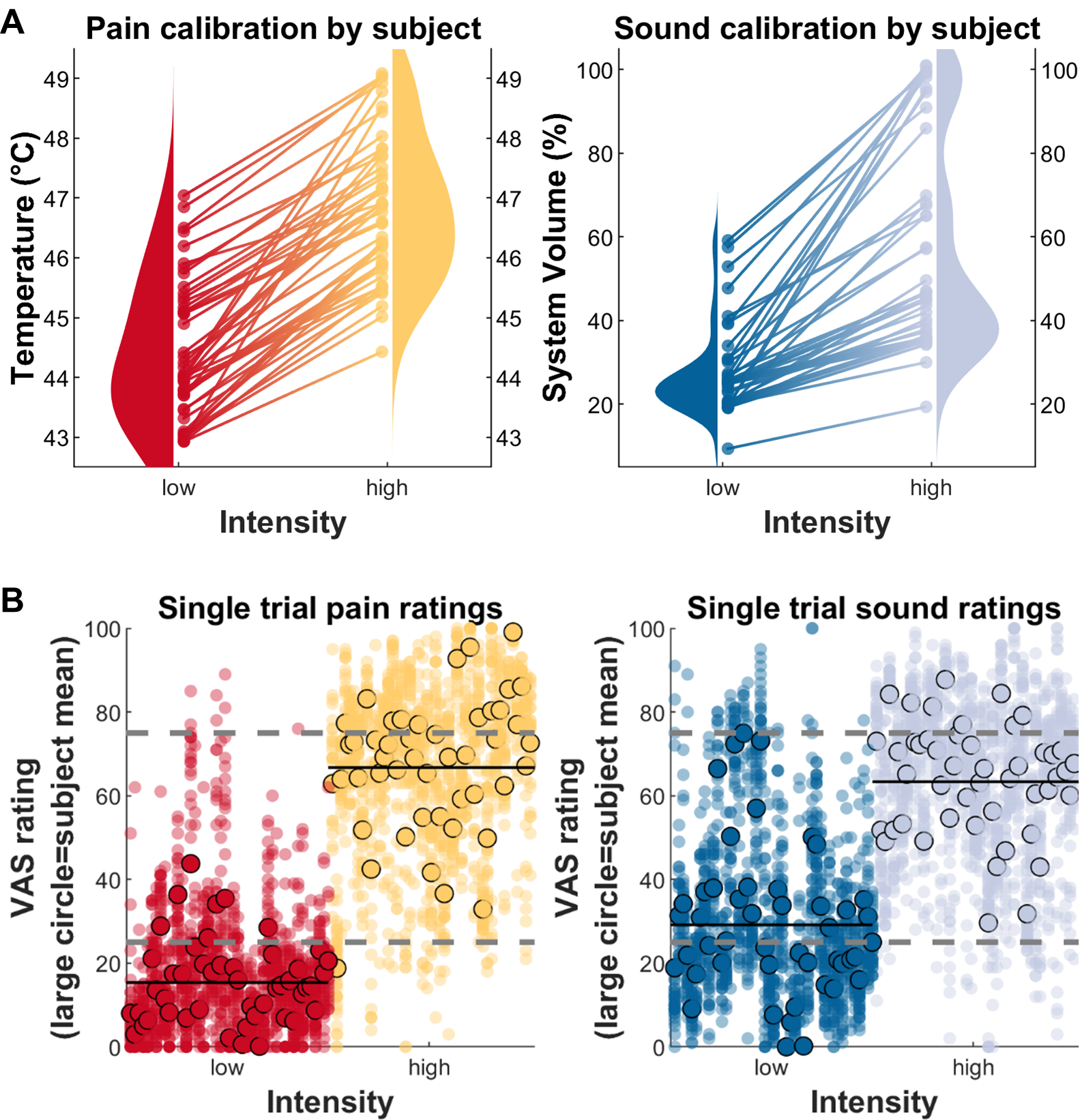

Supplement: S2 Fig — (A) Calibrated stimulus intensities corresponding to VAS 25 (low intensity) and VAS 75 (high intensity) for pain stimuli and sound stimuli. Each line represents the 2 intensities per modality per participant; the violin plots aggregate over participants. (B) Single trial ratings following pain stimulation and sound stimulation. Every column represents a single participant’s response to the respective intensity and modality; the bordered circle is a participant’s mean rating. The gray dashed lines is the “intended” rating as per calibration (VAS 25 for low and VAS 75 for high intensities). The black line is the actual mean rating over all participants. Data used to produce the figure can be found at https://www.doi.org/10.17605/OSF.IO/7JBV3. VAS, visual analogue scale. (TIF) [file pbio.3001540.s004.tif]

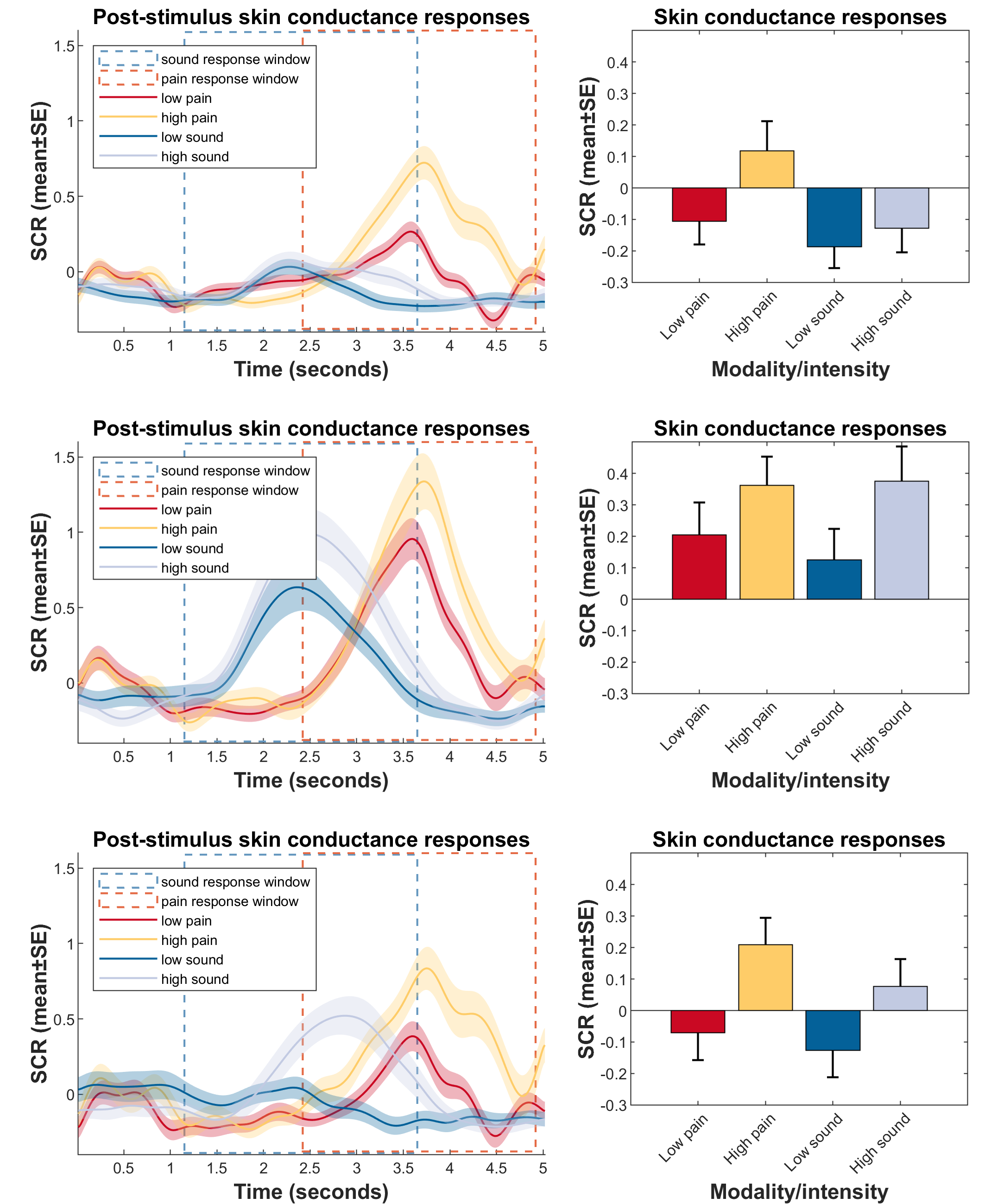

Supplement: S3 Fig — Rows show group means of SCR following no PE (row 1), intensity PE (row 2), and modality PE (row 3). Column show poststimulus SCR (left) and SCR averaged within the indicated response windows (right).Differences between conditions are largest in the no PE condition, smallest in the modality PE condition, which also shows the largest SCR amplitudes. Statistics of differences between conditions are displayed in S1 Table. All plots are based on log- and z-transformed data. Data used to produce the figure can be found at https://www.doi.org/10.17605/OSF.IO/7JBV3. PE, prediction error; SCR, skin conductance response. (TIF) [file pbio.3001540.s005.tif]

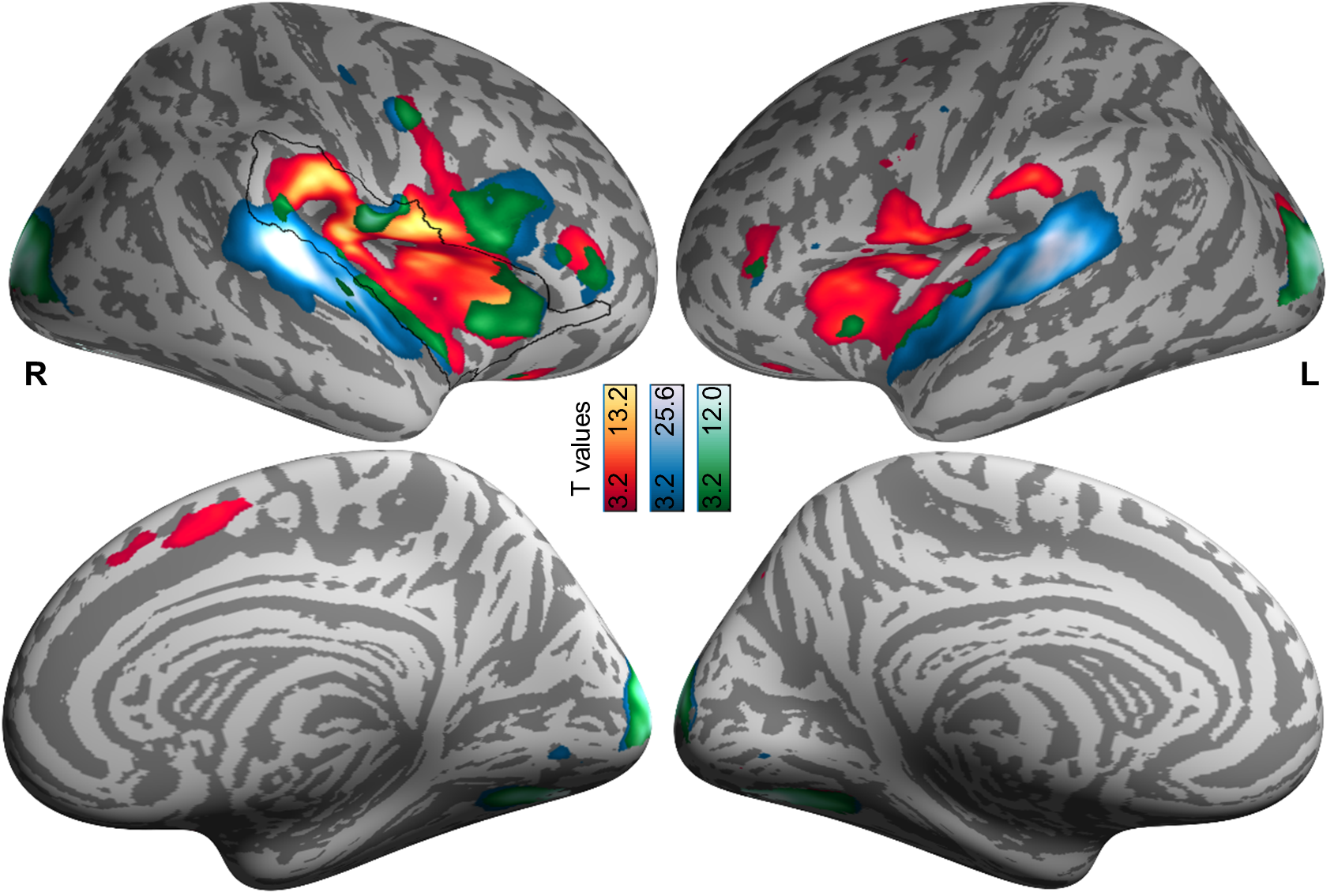

Supplement: S4 Fig — Activations are overlaid on an average brain surface and thresholded at p[uncorr.] < 0.001. The black line delineates the region of interest whose results are highlighted in Fig 5A and 5B. Data used to produce the figure can be found at https://www.doi.org/10.17605/OSF.IO/7JBV3. L, left hemisphere; R, right hemisphere. (TIF) [file pbio.3001540.s006.TIF]

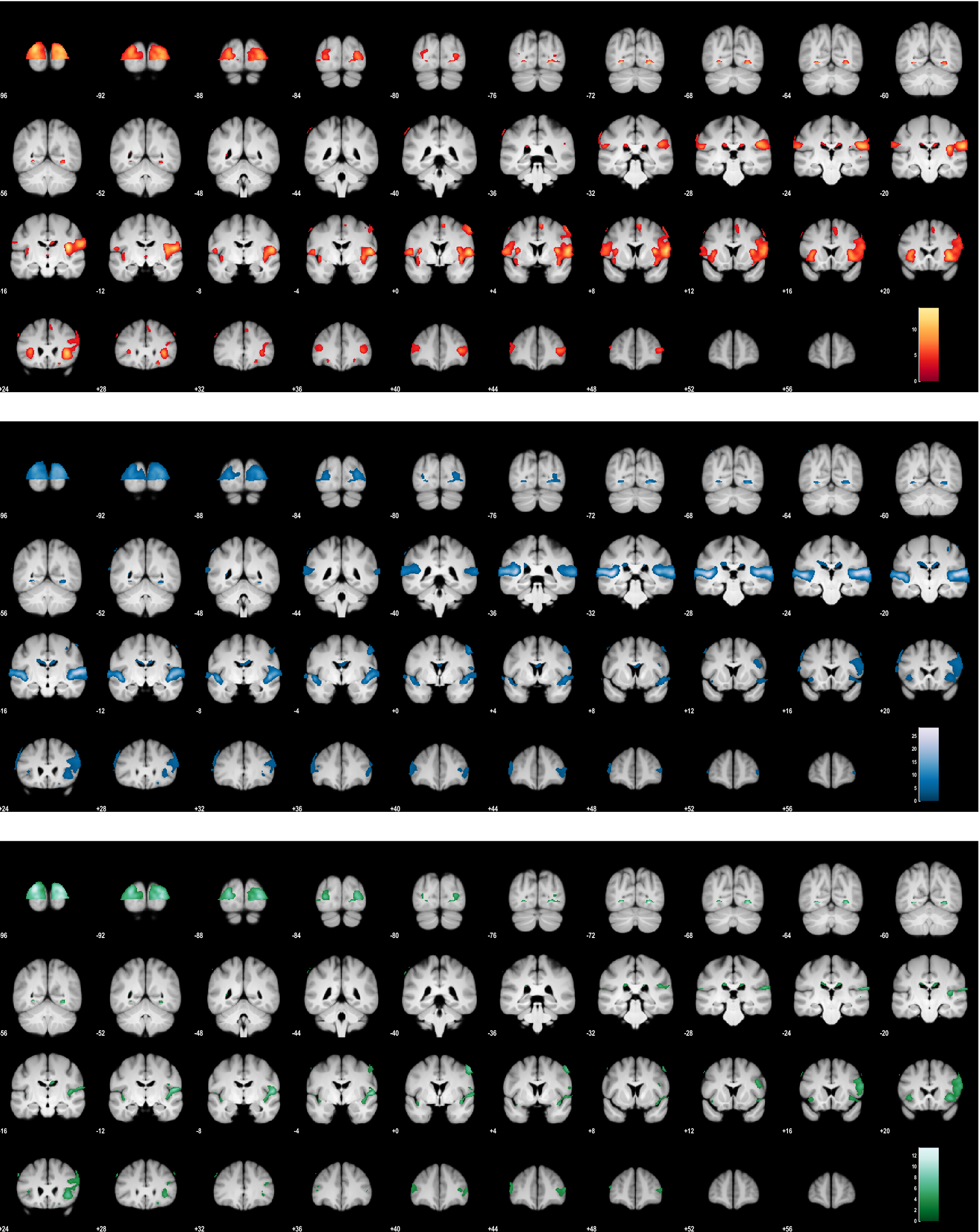

Supplement: S5 Fig — Activations are overlaid on an average brain volume and thresholded at p[uncorr.] < 0.001. (TIF) [file pbio.3001540.s007.TIF]

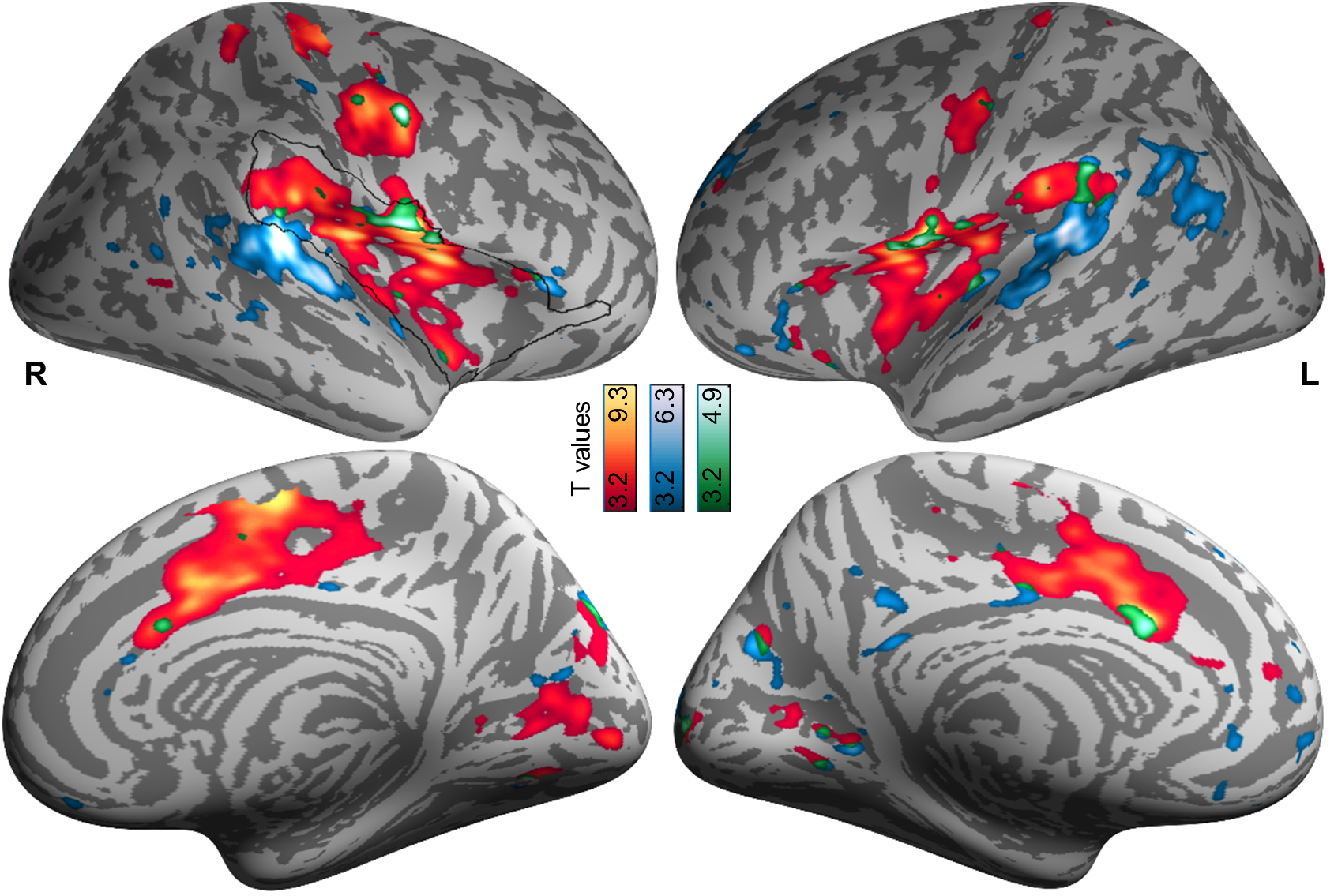

Supplement: S6 Fig — Activations are overlaid on an average brain surface and thresholded at p[uncorr.] < 0.001. The black line delineates the region of interest whose results are highlighted in Fig 5B and 5C. Data used to produce the figure can be found at https://www.doi.org/10.17605/OSF.IO/7JBV3. L, left hemisphere; R, right hemisphere. (TIF) [file pbio.3001540.s008.TIF]

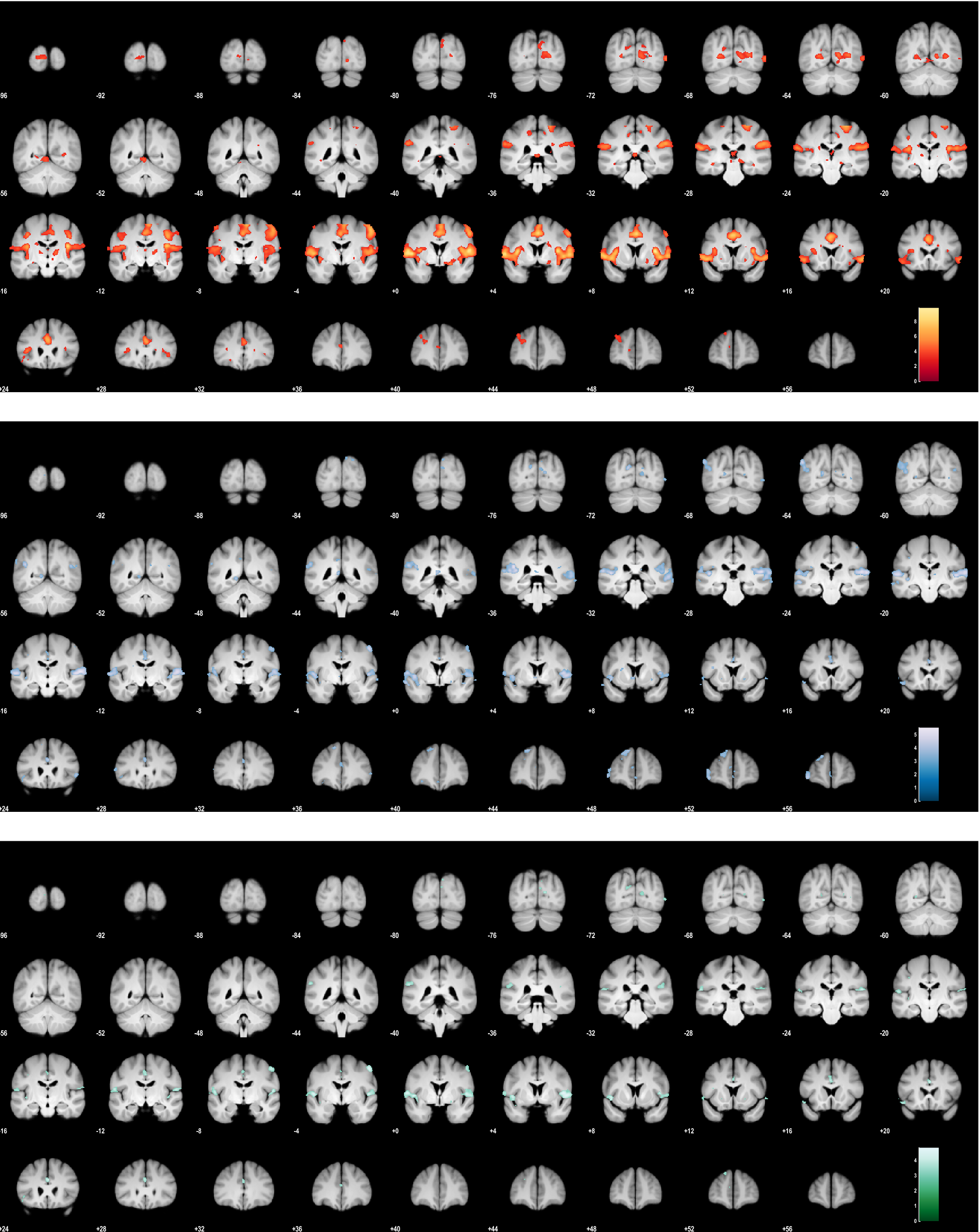

Supplement: S7 Fig — Activations are overlaid on an average brain volume and thresholded at p[uncorr.] < 0.001. (TIF) [file pbio.3001540.s009.TIF]

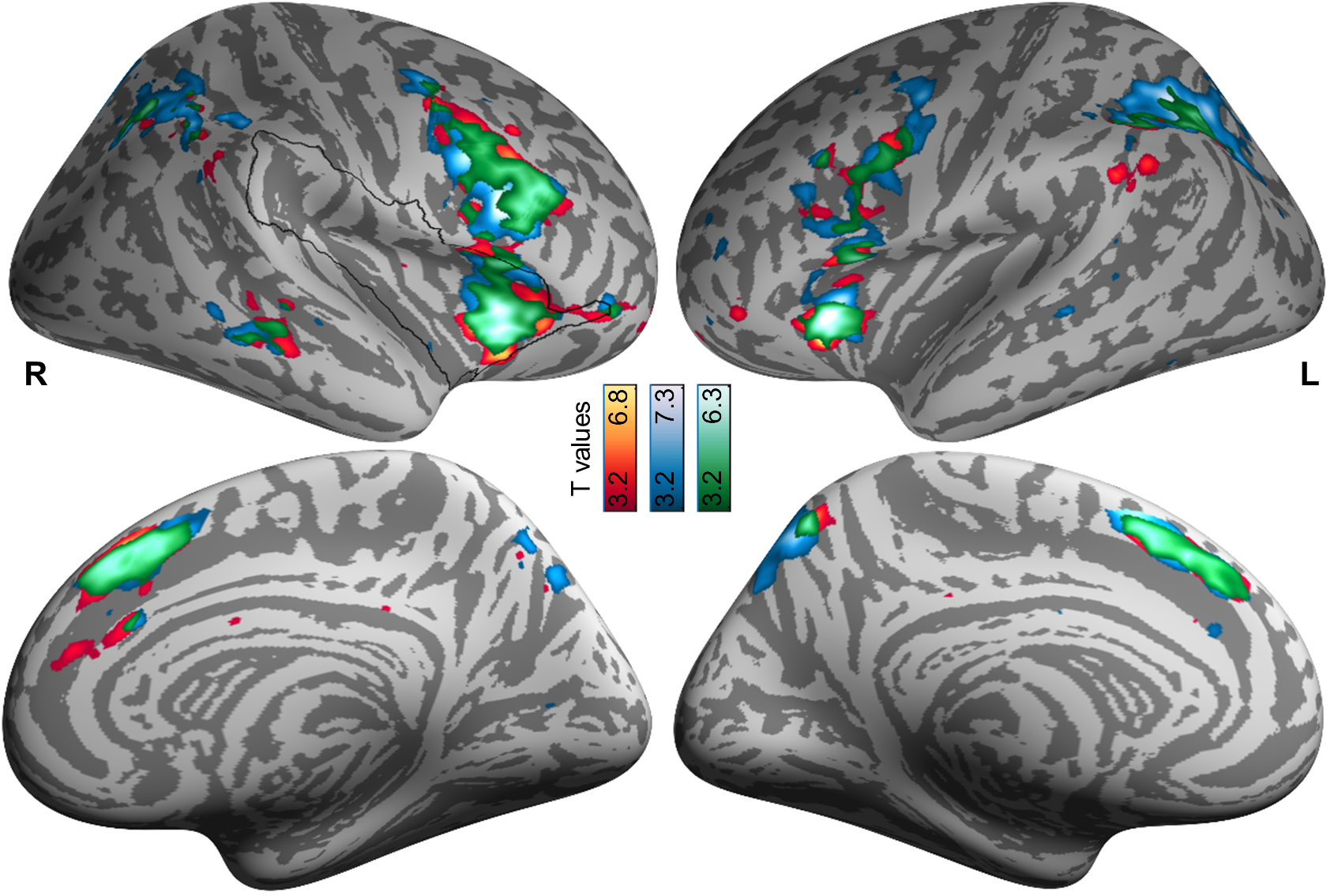

Supplement: S8 Fig — Activations are overlaid on an average brain surface and thresholded at p[uncorr.] < 0.001. The black line delineates the region of interest whose results are highlighted in Fig 6. Data used to produce the figure can be found at https://www.doi.org/10.17605/OSF.IO/7JBV3. L, left hemisphere; R, right hemisphere. (TIF) [file pbio.3001540.s010.TIF]

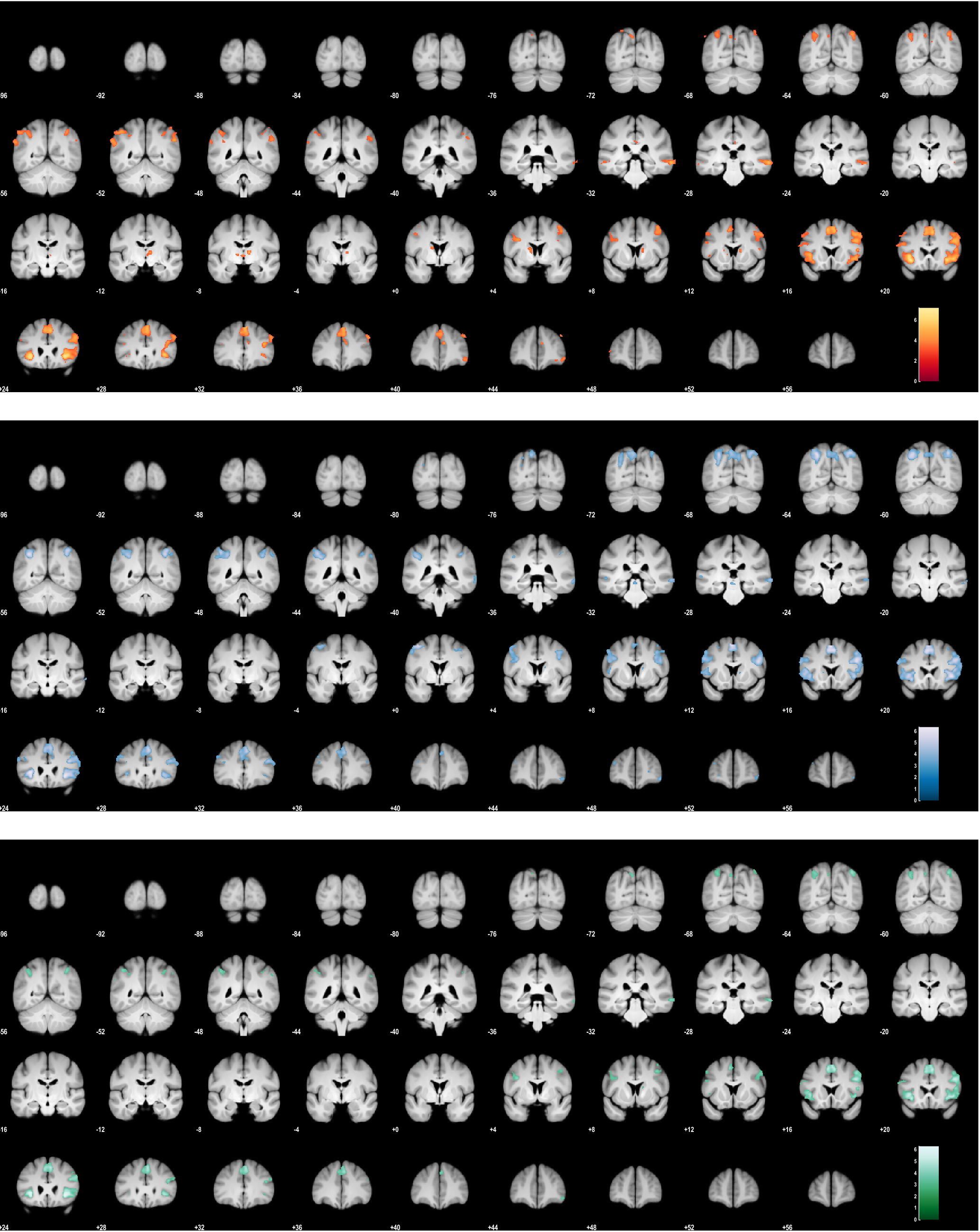

Supplement: S9 Fig — Activations are overlaid on an average brain volume and thresholded at p[uncorr.] < 0.001. (TIF) [file pbio.3001540.s011.TIF]

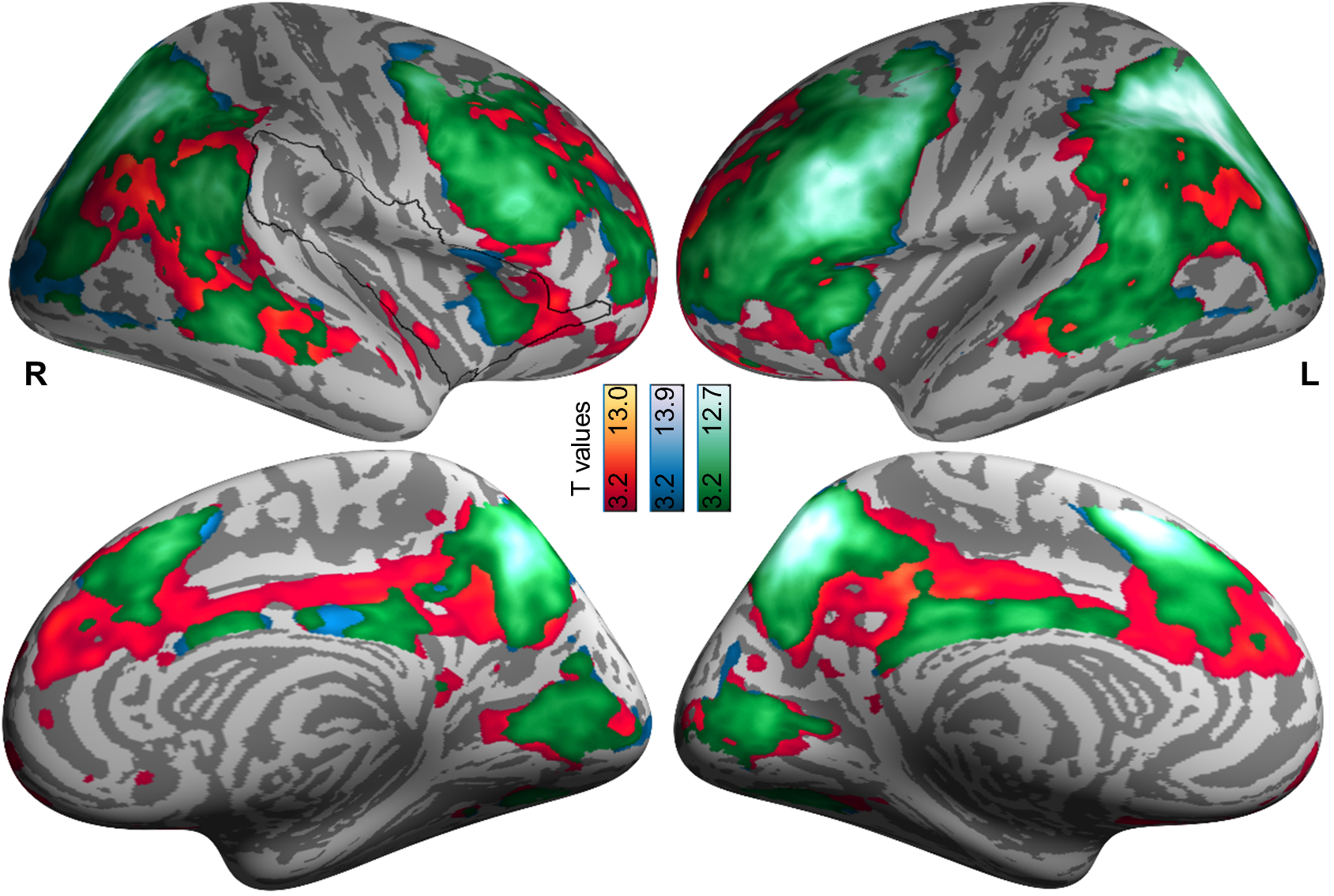

Supplement: S10 Fig — Activations are overlaid on an average brain surface and thresholded at p[uncorr.] < 0.001. The black line delineates the region of interest whose results are highlighted in Fig 7. Data used to produce the figure can be found at https://www.doi.org/10.17605/OSF.IO/7JBV3. L, left hemisphere; R, right hemisphere. (TIF) [file pbio.3001540.s012.TIF]

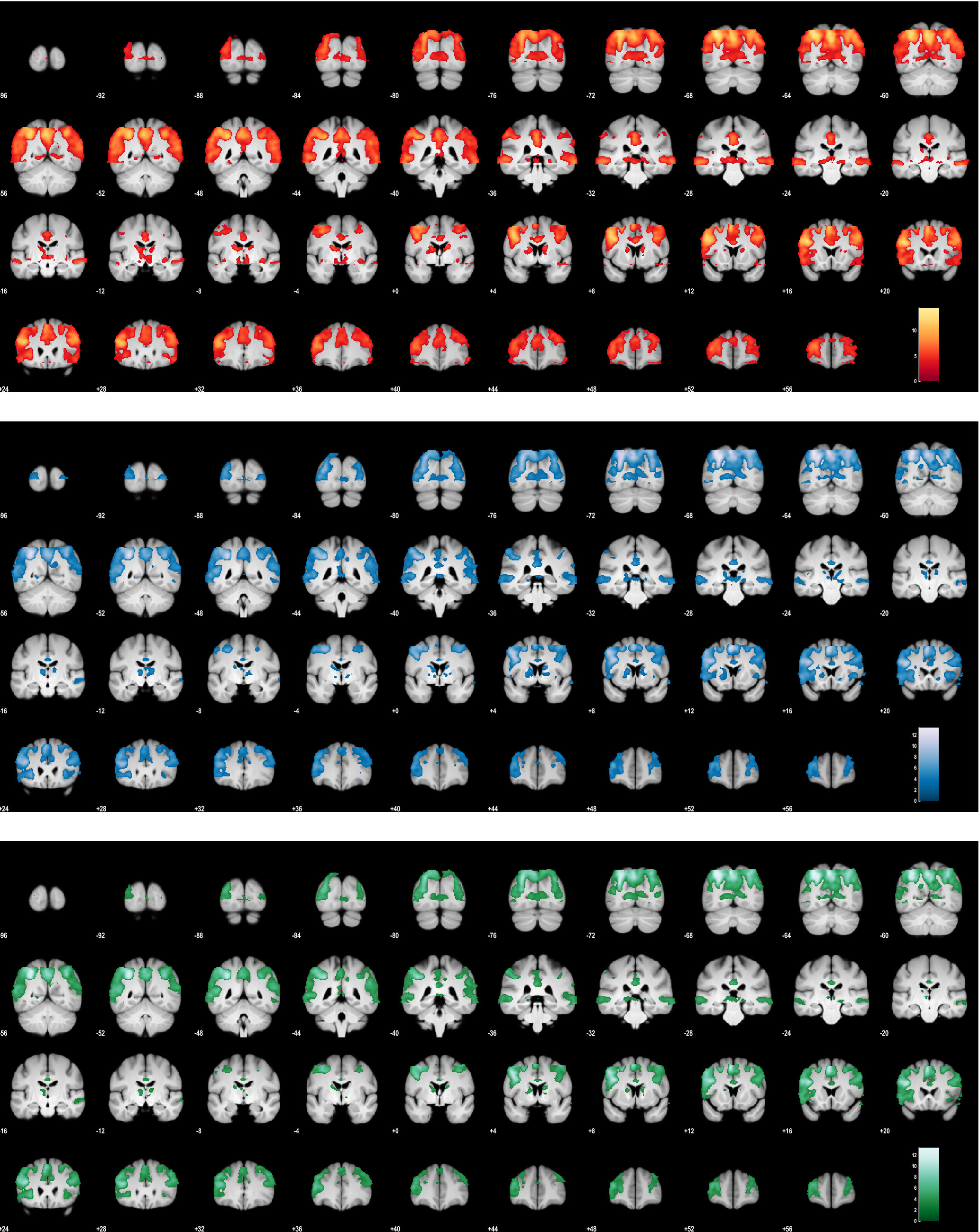

Supplement: S11 Fig — Activations are overlaid on an average brain volume and thresholded at p[uncorr.] < 0.001. (TIF) [file pbio.3001540.s013.TIF]

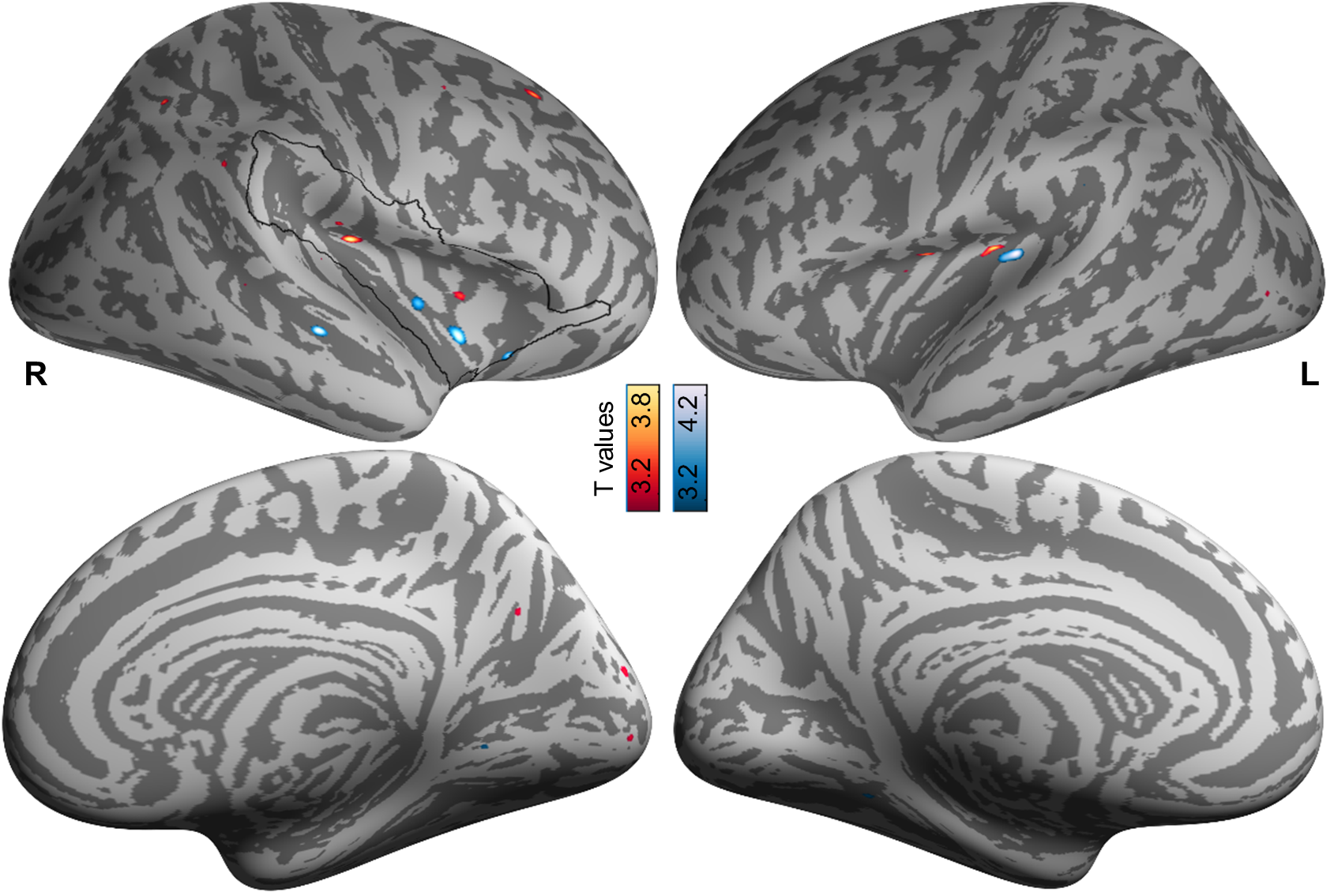

Supplement: S12 Fig — No significant conjunction activation prevails. Activations are overlaid on an average brain surface and thresholded at p[uncorr.] < 0.001. The black line delineates the region of interest whose results are highlighted in Fig 9. Data used to produce the figure can be found at https://www.doi.org/10.17605/OSF.IO/7JBV3. L, left hemisphere; R, right hemisphere. (TIF) [file pbio.3001540.s014.TIF]

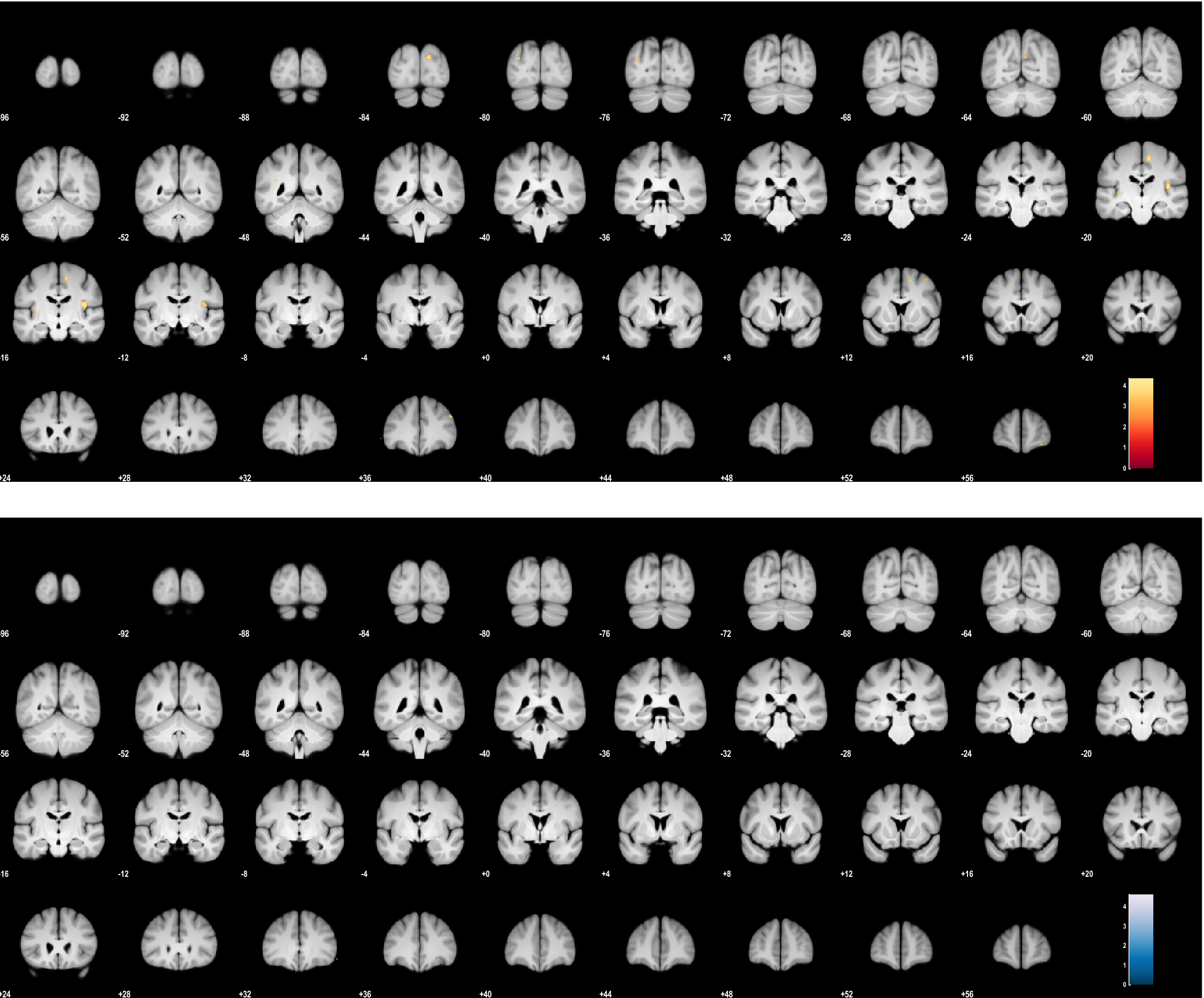

Supplement: S13 Fig — No significant conjunction activation prevails. Activations are overlaid on an average brain volume and thresholded at p[uncorr.] < 0.001. (TIF) [file pbio.3001540.s015.tif]

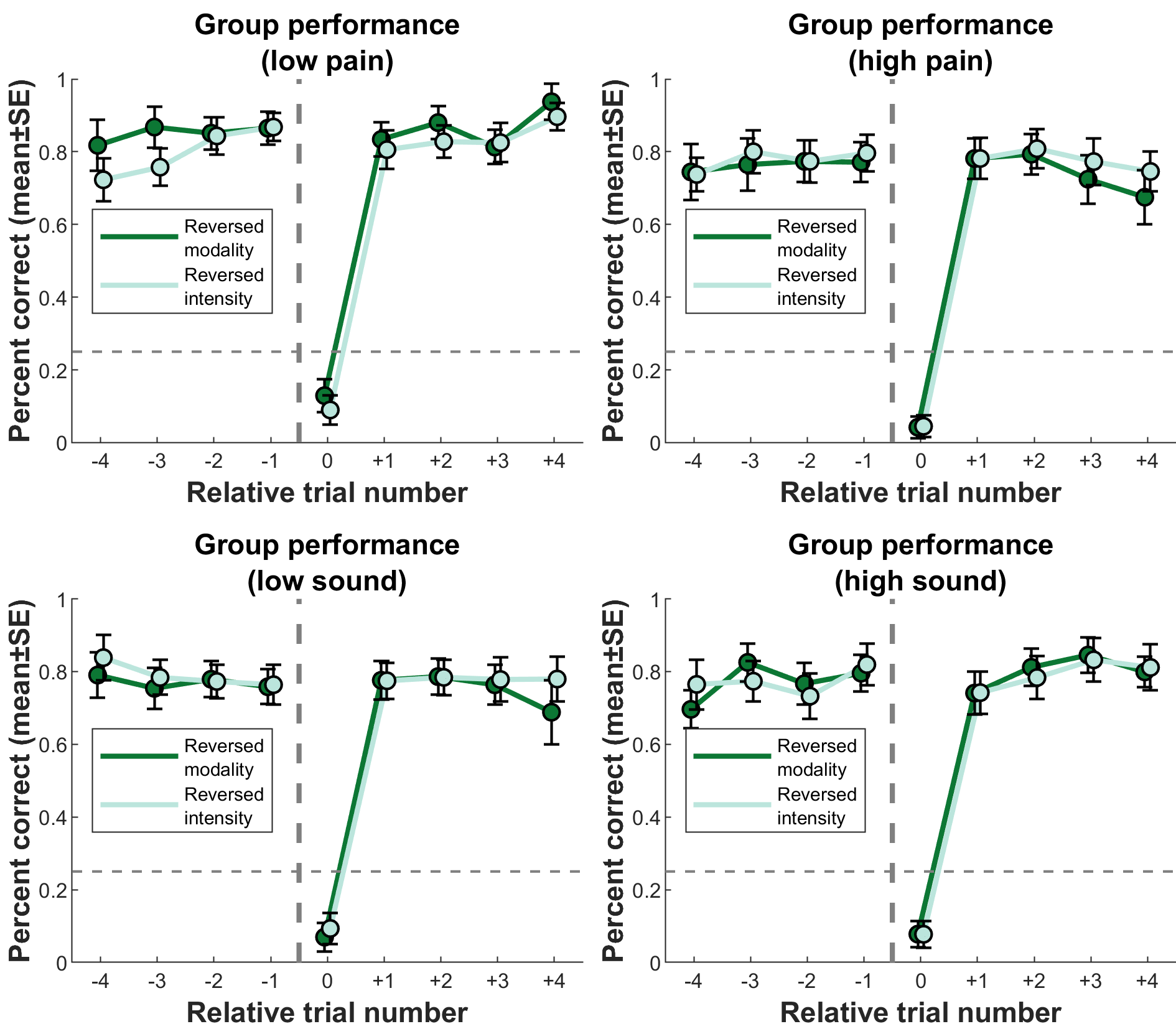

Supplement: S14 Fig — Grand mean performance is shown in Fig 3B. Data used to produce the figure can be found at https://www.doi.org/10.17605/OSF.IO/7JBV3. (TIF) [file pbio.3001540.s016.tif]
