# Supplementary material for: The human insula processes both modality-independent and pain-selective learning signals
Source: PLoS Biol. 2022 May 6;20(5):e3001540. doi: 10.1371/journal.pbio.3001540 (PMC9116652; doi:10.1371/journal.pbio.3001540)
Supplement: Attachment — Submitted filename: reviews_R1.pdf [file pbio.3001540.s017.pdf]

REVIEWERS' COMMENTS:

Reviewer #1:

In this manuscript, the authors present the results of a brain imaging study examining the brain representation of two types of prediction errors : 1) prediction errors pertaining to the intensity of the stimulation, and 2) prediction errors related to the modality of the stimulation (auditory or thermal). In order to generate these two types of prediction errors, they designed a very clever and elegant experiment where the outcomes predicted by predictive cues could either change on the intensity dimension (from low to high or from high to low), or on the modality dimension (from auditory to thermal or thermal to auditory). I didn't notice any important flaws in study design or analyses. Since this is to my knowledge the first study investigating modality prediction error, I would recommend publication. Still I have a relatively long list of minor issues, which I think should be addressed.

>> Thank you very much for your kind assessment. We have considered each of your comments and have changed the manuscript accordingly. All changes in the manuscript are highlighted in the "tracked changes" version of the manuscript (sections copypasted during discussion restructuring were underlined), for your convenience.

1. To me, the key aspect of the study are the *modality prediction errors*. This should be made clearer in the introduction. More specifically, why is it important to study these modality prediction errors? Are there concrete examples where the modality of the stimulation abruptly switches like in their experiment?

>> We agree that modality PEs are an exciting and understudies phenomenon. We have now included a respective paragraph in the introduction and expanded on the topic in line of (e.g.) your comment #2.

2. Related to the above point, I don't understand why the authors make the prediction of a weaker PE for switches in modality « in this case we expected a weaker signal, as the intensity - and thus salience and other general aspects - are intendedly not different between the expected and the received US. ». Modality PEs represent a *qualitative change*… I thought they would be even more surprising… That being said, the probability of intensity or modality changes is the same. Based on that, I wouldn't expect much variability in the strength of the PE. But I don't think that the amplitude of the PE is the right way to frame the hypotheses. What is more interesting are the structures involved. In the case of modality, there is the possibility that the PE could involve sensory and spatial orientation systems.

>> We were ultimately surprised by the extent of the effect of said qualitative change, which also turned out to dominate the SCR results. Following your suggestion, we have integrated the alternative hypothesis in the introduction. In the discussion, we also added emphasis on the overall absence of (cortical) structures differentially encoding pain versus sound modality prediction errors as suggested by Figure 7 and Supplementary Figure 10&11 (Paragraph starting with "Notably, activation following modality PEs…"). Given the reported findings, we did not separately report results from the respective contrasts (Pain_modPE>Sound_modPE and Sound_modPE>Pain_modPE) as in fact no clear picture emerges beyond what is shown in the supplement.

3. The rationale for focusing on the insula is also not clear. It feels like it would bias the results to only reveal unsigned and modality-independent PEs. It also produces an asymmetry between auditory (processed in STG, which is not in the mask) and thermal stimuli (processed in posterior insula and

parietal operculum, which are within the mask). If I personally had to choose a priori regions, I would have focused on 1) "salience" regions (aINS AND ACC), 2) sensory regions (OP, dpINS, S1, STG), and 3) spatial orientation fronto-parietal regions. I have no doubts that a case can be made for the entire insula, but the paper would be stronger if the authors could present a *brief theory of insula function* in the introduction.

>> Our reasons for restricting analyses to an extended insula mask include a precedence from previous pain studies (in our lab and others) showing critical involvement of the insula, and the candidate role of the insula as multimodal integration area. We agree that several aspects of our paradigm (stimulus characteristics, task demands etc) may also be reflected elsewhere; since our focus is decidedly on pain and PEs however, we feel that the inclusion of whole-brain results still permits a fully transparent assessment of those areas while preserving sensitivity and a coherent narrative for insula-related functions. We have now laid out this argument more clearly and included more background information concerning insula functions in the introduction

4. I also think that I would have preferred an introduction that leaves more room to the possibility of signed, not just unsigned, prediction errors. On the other hand, I also understand the need to tailor the introduction to the main findings of the experiment. Still, I think I would have preferred *an introduction that raises the possibility of signed prediction errors*, and then a discussion as to why they perhaps didn't came out as strongly as expected? This is more a remark than a criticism. I leave that entirely up to the authors.

>> We added emphasis on this aspect in the introduction. In the discussion, we have now added a comment on the size of the signed PEs and speculate that they may be under-represented due to overlaps with intensity-related activation (cf your comment #8). (paragraph following “Due to the task-inherent structure…”)

5. Modality PEs are confounded with spatial location. I don't think that this is a problem here, but I think it should be discussed briefly. It could be eventually nice to have an experiment that tests spatial location within-modality.

>> We agree that location is one of those “non-specific” parameters that ideally should be controlled for. However, there are technical constraints, and in this case, the comparator (i.e. auditory) is non-somatosensory, without a strict “location” on the body. Nevertheless, we have now added the idea of a within-modality condition to control for location on the body (e.g. painful stimulation on the ear). (paragraph “Regions coding for aversiveness per se…”)

6. I don't think that subjective intensities were perfectly matched. "100 = unbearable pain" probably refers to an absolutely stronger sensation than "*100 = extremely unpleasant*". Cross-modality matching would have been a better psychophysical technique here. But as I said previously, I don't think that the magnitude of the PE is particularly important here. This is more a remark than a criticism. The authors should simply be careful not to over-state the matching in absolute intensity.

>> We have actually reported this erroneously, thank you for spotting this. The respective anchors read “unbearably painful” (German “unerträglicher Schmerz”) versus “unbearably unpleasant” (German “unerträglich unangenehm“). Also, we like to point out that the matching-by-intensity is merely a proxy for the desired matching-by-salience/arousal. While this matching was incomplete, we discuss the implications of this (discussion paragraph stating “Although we aimed to perfectly match salience

between stimulus modalities"). We have added a comment concerning cross-modality matching, which we think is difficult due to residual phenomenological differences between the modalities, and importantly, the risk of hearing loss in overly intense sound stimulation.

7. There is a potential problem with the use of parametric modulators to track PEs. For instance, the parametric modulator for unsigned intensity PE (-1: lower than expected; 0: no change; 1: higher than expected) could be - for instance - entirely driven by a very strong difference between higher than expected and no change, with no difference between smaller than expected and no change. A stricter approach would have be to look at the *conjunction of (lower than expected > no change) and (higher than expected > no change)*. The same principle applies to modality PEs and signed PEs, and skin conductance responses. I think the findings would be stronger if the authors could show that the same findings hold when using this stricter criterion. However, I am willing to accept the parametric modulators if this is only what comes out as significant.

>> This is an interesting point as it in theory applies to every regression analysis, where you could rephrase differences between values as pair-wise conjunctions of neighboring regressor values. However, this would decrease sensitivity, as a conjunction applies more conservative criteria for assessing significance. In spite of this concern, we have now performed such an analysis (splitting PEs by PE+ and PE-) using the locations identified by the full analysis. The z values from this adjunct analysis are now reported in the results on signed intensity PEs to flesh out the relative strength of the contributions below and above zero. We also report uncorrected p values; however, these do not survive correction for multiple comparisons.

8. Prediction errors were mostly (only ?) observed for the first « reversal » trial. This is not a problem in and of itself, but I think that this might have reduced the power to observe statistically significant effects. Perhaps a better approach would have been to have the transitions more probabilistic, rather than all-or-none. This is just a remark, not necessarily a problem that the authors need to assess in their manuscript.

>> This is a good point. When designing the experiment we were anxious that volunteers can learn the contingencies of two CS with four possible US in the context of frequent reversals. Pilot data outside the MR scanner (not included in the paper) did not lead us to expect that many one-trial learning results. However, even in the main sample, not all volunteers performed that well, actually some learned very slowly, which suggests that the design complexity was a good compromise. We have included a paragraph in the discussion to this regard, as this was also noted by reviewer #2.

9. It seems like SCR from all participants were lumped together as if originating from one very long experiment in a single subject (or in a « fixed effects » manner). Ideally, the authors should have used summary statistics from every participant, or even better a hierarchical regression model taking into consideration within and between-subjects effects. The problem is that « fixed effects » can be driven by a few participants and not reflect group effects. The paper would be stronger if the results came out as significant using an approach that takes into consideration between-subjects variability. That being said, analyses of SCRs could be an exception to this general principle given the difficulties in dealing with non-responders. If that is the case, maybe the authors could justify this methodological choice a little bit better in the methods section. What stroke me as a bit odd were the relatively high p-values, which I think should be interpreted with caution. Note that I don't think that SCR effects are a « sine qua non » condition for interpreting brain results.

Arguably, SCRs could be more noisy than brain signals. But I agree that starting with SCR results tell a better story.

>> We are sorry for the misunderstanding here. We actually employed a linear mixed model considering all available levels of measurement. We have now clarified this and are describing the analysis as "random intercept linear mixed model predicting each subject's and each trial's SCR".

10. Figure 4D indicates that SCRs for modality and intensity PEs have the same magnitude. This is contradictory with what can be seen in figure 4C. Why the discrepancy?

>> We see how this perception can arise; however, we point out that the analyses are in fact based on different trials. The second analysis is only considering the position relative to the reversal, not whether a PE took place. We have now elaborated this in the legend "Within trials, data is pooled over all modalities, intensities and expectations, i.e. does not consider whether subjects correctly predicted the subsequent stimulus"

11. First paragraph of imaging results: I would have expected the authors to report STG findings *in the text* as well.

>> We have corrected this omission, which also stems from the intention to focus on pain results mentioned elsewhere ("Activation following sound stimulation peaks in the superior temporal gyrus (XYZMNI 65.9/-23.8/10.2, T = 25.7, p[corr. wb.] ≈ 0), just outside the extended insula mask.").

12. Why call the effects of the stimulus « onset » in the figures. Was only the onset of the stimulations modelled?

>> Inadvertently, we have retained the terms used internally when discussing the results, where we use "onset" as jargon to refer to "stimulus-onset-related activation" as opposed to their parametric modulators. We now more accurately use the term "main" and explain it to refer to the main effect of the respective modality (note that we have also changed "VAS" to "ratings", for better legibility).

13. I found the discussion to be too defensive and focusing on methodological considerations. It would be preferable to at least start by explaining why the key findings of the experiment are interesting. This parallels my comments on the introduction.

>> We have restructured the discussion accordingly (i.e. discussion/contextualization of results first, then methodological concerns) to shift focus. Since we have also incorporated multiple comments from reviewer 2, the discussion now includes more content-related aspects.

Despite my perhaps long list of comments, I'd like to re-state that this is a well-designed and well-conducted study. Findings of unsigned modality-independent PEs in the aINS are interesting and novel. Examining modality-related PEs is novel and original, and the paper is likely to get cited because of that. There still are a few imperfections (see above) - notably experimental effects might not have always come out as strongly as desired - but that doesn't undermine the originality of the study and its main findings. The introduction and discussion could be substantially improved, but this is well within the author's reach.

>> Thank you again for your positive assessment.

Reviewer #2:

>> We thank you for your thorough assessment of the article. We have taken care to address your concerns one by one. All changes in the manuscript are highlighted in the "tracked changes" version of the manuscript (sections copypasted during discussion restructuring were underlined), for your convenience.

Major comments.

1. To distinguish aversiveness from saliency, I wondered whether the experimental design could have included an appetitive condition. Could this be discussed in comparison to studies with different designs comparing reward from punishment learning signals since they also report a critical role of the anterior insula during punishment avoidance learning and prediction errors signals in fMRI (Pessiglione et al., Nature 2006), intracranial recordings (Gueguen et al., Nat. Comm 2021) and lesion studies (Palminteri et al., Neuron 2011).

>> Very interesting, an appetitive condition would indeed make a good contribution. We are now discussing this aspect, including the referenced literature, mostly in terms of future directions.

2. Relatedly, although there is a paragraph commenting study design difference between probabilistic RL tasks and the pavlovian approach used in this study, I think further discussing the advantages of the proposed paradigm (with binary signals) over parametric designs (with more graded expectation/outcome values) to study the PE. The entire paper is framed on the concept of PE but the experiment seems not optimal to examine fine-grained PE signals (signed and unsigned PE signals which were sub-sampled by the experimental structure) or even to check that the PE signals are "truly prediction error signals" according to a formal/axiomatic approach. I might have missed critical aspects of the paradigm or in the statistical analyses made (see below).

>> Reviewer 1 has also pointed this out, and we agree that the high performance was surprising at first. When designing the experiment we were initially anxious whether volunteers can learn the contingencies of two CS with four possible US, but pilot data outside the MR scanner (not included in the paper) did not lead us to expect that many one-trial learning results. However, even in the main sample, not all volunteers performed that well, actually some learned very slowly, which suggests that the design complexity was a good compromise. We have included a paragraph in the discussion to this regard that a more granular assessment of PEs may be possible with a carefully modified design. The advantages of the protocol are highlighted in the same paragraph.

3. I could not figure out exactly what was done in terms of fMRI data analyses: in the figures 5-9, authors label their regressors (VAS, PE, modPE, modality PE (repeated twice in fig5 legend, please correct, …). I could not find a clear description of these regressors so that I had to guess what was done exactly (there are 7 lines of text page 23 to explain what was done to generate figs 5, 6, 7, 8, 9). Relatedly, I could not find which time window within the task was used to perform the fMRI analyses (the US period?). For instance, when investigating VAS (stimulus perception), fMRI could be either triggered by the US stimulus or by the VAS rating onset.

>> Please excuse us for being so unclear about this. We have added a more thorough description of the employed GLMs. We have restructured the Figure 5 legend.

4. Unpleasantness/Pain ratings: in the paper it seems that unpleasantness signals are domain specific (activating modality specific brain regions); other groups report modality independent unpleasantness signals, notably in the anterior insula (e.g., Corradi del Acqua et al 2016) ?. This issue (modality specific vs. independent of unpleasantness) *could be discusses*.

>> We have added a section to the discussion addressing this issue. Please note that we do in fact find an overlap in regions known to encode unpleasantness (specifically, the central operculum and the ACC), but we now include studies reporting a larger overlap and speculate on the reasons for the divergence.

[4(2)] I also wondered whether using "only" four types of unpleasant stimuli (low vs. high pain and low vs. high sound) could be an issue compared to paradigms generating perhaps more variable ratings?

>> This could indeed be an interesting aspect to entertain going forward. However, with the current paradigm, increasing task difficulty e.g. by adding more stimulus categories may have compromised performance and may have led to substantial performance-related exclusions.

5. Minor points:
5.1 I would also be interested if the authors could articulate clearly the results of this study and their own previous work in which they conclude that anterior insula pain expectation /PE signals are not related to aversiveness (Fazeli & Buchel 2018).

>> We have elaborated this alongside the issues raised in your Major comment #1 and #4(1) by expanding the discussion to cover behavioral/reinforcement systems (appetitive versus aversive/reward versus punishment) as well as aversiveness/unpleasantness as a supramodal construct.

5.2 In the behavioral analyses, I suggest to use a more classic ANOVA followed by post-hoc test instead of using multiple t-tests to compare the conditions

>> The analyses pertaining to our SCR results were indeed insufficiently introduced. We have amended this by pointing to the fact that we have employed linear mixed effects models (not t-tests as the statistic might have falsely suggested). As for the performance analysis, we have now added such a model using average peri-trial performance to demonstrate significant differences of the reversal trial to all other trials.

5.3 could you check throughout the manuscripts the terminology chosen to explain each concept and check for consistency across sections to reduce possible reader's confusion (e.g., for VAS pain/unpleasantness ratings, these are interpreted as a proxy of "stumulus perception" and/or "intensity effects";

>> Thank you for spotting this. We have unified the verbiage in several spots (mostly making clear that pain intensity refers to pain ratings); also, we have changed the labels for the fMRI signal bar graphs throughout that they now refer to “main” and “rating” effects (previously “onset” and “VAS”).

5.4 Figures legends: please define the meaning of black (continuous vs. dashed lines) rectangles (does this corresponds to significant regerssors ?)

>> Apologies for the oversight, we have now added the explanation in each legend, “[In the fMRI signal bar graph,] Black rectangles highlight the regressors used for analysis; solid line indicates analysis with the respective individual regressor, dashed line indicates conjunction analysis.”
